# Influence of Previous Therapy for Neutropenia Caused by Combination Therapy of Ramucirumab and Docetaxel

**DOI:** 10.3390/cancers16112076

**Published:** 2024-05-30

**Authors:** Hiroyuki Ohno, Takahiro Hayashi, Shota Torii, Miduki Niwa, Nanae Katagiri, Yuri Nakao, Shota Mano, Norio Takimoto, Tomoyuki Hirashita

**Affiliations:** 1 Department of Pharmacy, Gifu Prefectural General Medical Center, Gifu 500-8717, Japan; h.oh-no@outlook.jp (H.O.); nedobedosaikou@gmail.com (S.M.); hirashita-tomoyuki@gifu-hp.jp (T.H.); 2 College of Pharmacy, Kinjo Gakuin University, Nagoya 463-8521, Japan; 3 Department of Pharmacy, Kariya Toyota General Hospital, Kariya 448-8505, Japan; toriishota1007@gmail.com (S.T.); norio.takimoto@toyota-kai.or.jp (N.T.)

**Keywords:** combination therapy of ramucirumab and docetaxel, neutropenia, immune checkpoint inhibitor, cytotoxic chemotherapy

## Abstract

**Simple Summary:**

Ramucirumab (RAM) + docetaxel (DTX) therapy is recommended as a second-line treatment for non-small cell lung cancer (NSCLC) and pretreatment with immune checkpoint inhibitors (ICIs) has been reported to improve the therapeutic efficacy of this therapy. Meanwhile, caution should be exercised for the development of febrile neutropenia during this treatment and preventing the onset of neutropenia while continuing the treatment is greatly important. This study found that previous treatment with ICIs reduced the incidence of grade ≥ 3 neutropenia after RAM + DTX therapy in patients with NSCLC, regardless of the influences of pretreatment with cytotoxic anticancer agents. A history of ICI treatment may have a similar influence on carcinomas other than NSCLC.

**Abstract:**

In the present study, the influence of previous immune checkpoint inhibitor (ICI) therapy with ramucirumab (RAM) + docetaxel (DTX) therapy on the occurrence of severe neutropenia in patients with non-small cell lung cancer (NSCLC) was evaluated, taking into account the influences of cytotoxic chemotherapy used in pretreatment. The study participants included patients who received a combination therapy of RAM and DTX as cancer chemotherapy for NSCLC. The influences of previous ICI treatment and pretreatment with cytotoxic anticancer agents on the development of grade ≥ 3 neutropenia were analysed. A total of 89 patients, including 50 with and 39 without a history of ICI treatment, were analysed. Kaplan-Meier curves showed a significant difference in the influence of previous ICI treatment on the development of grade ≥ 3 neutropenia (*p* = 0.006). Moreover, Cox regression analysis identified a history of ICI treatment and prophylactic administration of G-CSF as factors associated with the development of grade ≥ 3 neutropenia (*p* = 0.018 and *p* < 0.001, respectively). This study found that previous treatment with ICIs reduced the incidence of grade ≥ 3 neutropenia after RAM + DTX therapy in patients with NSCLC, regardless of the influences of pretreatment with cytotoxic anticancer agents.

## 1. Introduction

A large multicentre randomised trial comparing supportive care alone with supportive care plus cisplatin-based chemotherapy has demonstrated the survival benefits of cisplatin-based chemotherapy in advanced non-small cell lung cancer (NSCLC) [1]. Thus, cisplatin-based chemotherapy has been used proactively for NSCLC. Meanwhile, the REVEL trial comparing docetaxel (DTX) alone with combination therapy of DTX plus ramucirumab (RAM) has demonstrated that RAM + DTX therapy prolonged both overall survival (OS) and progression-free survival (PFS) compared with DTX alone [2,3]. An exploratory analysis of the REVEL trial demonstrated that RAM + DTX therapy as a second-line therapy had no effects on OS and PFS, regardless of the type of first-line therapy. Thus, they have reported that survival benefits from adding RAM to DTX were not compromised by previous treatment with bevacizumab [4]. Accordingly, the 2022 Japanese clinical practice guidelines for lung cancer—including malignant pleural mesothelioma and thymic tumours—have recommended DTX + RAM therapy as a treatment option for second- and later-line therapy. They also reported that grade ≥ 3 hypertension, neutropenia, febrile neutropenia (FN) and leukopenia (squamous cell carcinoma only) occurred more frequently with RAM + DTX therapy than with placebo + DTX therapy [4]. In the National Comprehensive Cancer Network (NCCN) Clinical Practice Guidelines in Oncology—Myeloid Growth Factors—Version 1 [5], the following risk factors for the induction of FN are identified: older age (particularly ≥65 years), chemotherapy or radiotherapy, history of neutropenia or bone marrow invasion, infection, wounded orifice, history of surgery, performance status, kidney failure and liver failure (particularly bilirubin deterioration).

Recently, immune checkpoint inhibitors (ICIs) have been introduced and various reports have documented their efficacy. In patients with advanced, previously treated squamous NSCLC, the OS, response rate and PFS treated with nivolumab have been significantly better than those treated with DTX, regardless of the PD-L1 expression level [6]. Therefore, the Japanese guideline recommends platinum-based chemotherapy plus ICI therapy, a combination of ICIs or ICI monotherapy as a first-line treatment for NSCLC. Recently, several studies have reported the influence of previous ICI therapy on the efficacy of subsequent treatment. A retrospective study on RAM + DTX therapy reported that the PFS in the presence of ICI pretreatment history was superior to that in the absence of ICI pretreatment history [7]. Molife et al. [8] have reported that sequential treatment with RAM after ICIs for patients with NSCLC can be expected to prolong OS. The use of nivolumab as the third- to sixth-line therapy may enhance subsequent chemosensitivity in patients with unresectable advanced or recurrent gastric cancer [9]. Several reports have indicated that a history of ICI treatment influences the incidence of adverse events in subsequent treatment. The prevalence rates of adverse events such as fever, myalgia, arthritis, pleural effusion and pneumonitis have been reported to increase in patients with NSCLC who received RAM + DTX therapy after having been previously treated with ICIs. This finding was attributed to the residual pharmacological effects of ICIs [7]. Other relevant findings include that the practical use of osimertinib in Japanese patients who received immediate previous nivolumab therapy frequently resulted in interstitial lung disease [10]. Furthermore, using osimertinib immediately after nivolumab significantly increased the frequency of grade ≥ 3 hepatotoxicity in patients with advanced NSCLC harbouring an epidermal growth factor receptor mutation who developed T790M resistance [11]. Thus, several reports [7,10,11] have argued that a history of ICI treatment has a negative impact on adverse events due to subsequent anticancer therapy in patients with NSCLC. Recently, the incidence of severe neutropenia caused by RAM + DTX therapy in patients with NSCLC with a history of ICI treatment was significantly lower than that in patients without a history of ICI treatment [12]. However, the number of patients in the previous study was insufficient because it was a single-centre study and the influence of previous ICI therapy on cytopenia due to cytotoxic chemotherapy used in first-line treatment could not be studied. Therefore, this multicentre study investigated the influence of previous ICI therapy on the development of severe neutropenia caused by RAM + DTX therapy, taking into account the influence of pretreatment with cytotoxic chemotherapy on cytopenia.

## 2. Materials and Methods

### 2.1. Data Source and Study Design

This retrospective cohort study was approved by the Epidemiology and Clinical Research Ethics Review Committee of Gifu Prefectural General Medical Centre (ethical approval number: 631, date: 9 February 2021), the Research Ethics Review Committee of Kariya Toyota General Hospital (ethical approval number: 711, date: 31 August 2021) and the Ethics Review Committee on Research In-volving Human Subjects of Kinjo Gakuin University (ethical approval number: R21014, date: 19 July 2021) and carried out in accordance with the approved guidelines. All study participants provided informed consent. Participants included patients who received a combination therapy of RAM and DTX as cancer chemotherapy for NSCLC at Gifu Prefectural General Medical Center and Kariya Toyota General Hospital between April 2016 and December 2020.

To administer the combination therapy of RAM and DTX, 50 mg diphenhydramine and 6.6 mg dexamethasone were orally and intravenously pre-administered, respectively, and 10 mg/kg RAM (1 h intravenous infusion) and 60 mg/m^2^ DTX (1 h intravenous infusion) were successively administered. The interval between two cycles of RAM and DTX therapies was 3 weeks. All study participants were recruited. However, those who temporarily or permanently stopped RAM, DTX or RAM + DTX therapy at any period were excluded from the analysis.

### 2.2. Data Collection

Patient data were collected from electronic patient files available in the databases of Gifu Prefectural General Medical Center or Kariya Toyota General Hospital.

At the start of treatment, the following variables were collected: age, sex, performance status, World Health Organization classification of NSCLC, cancer stage, planned relative dose intensity (RDI) (%) of RAM and DTX, detailed treatment history (past chemotherapy, presence of cytotoxic anticancer agent used just before regimen, duration that passed from using cytotoxic anticancer agent, history of platinum and history of taxane, radiotherapy career, recent operation career and history of ICI therapy) and the onset of grade ≥ 3 neutropenia immediately before chemotherapy. The RDI (%) for RAM and DTX was calculated using the following formula: actual dose/planned dose, i.e., 3.3 mg/kg/week and 20 mg/m^2^/week, respectively, multiplied by 100. The administration period for the planned dose was set to 3 weeks. Data on the following variables were extracted during the treatment period: body height; body weight; body surface area; course number of the combination therapy of RAM and DTX; dose interval of RAM and DTX therapies; RAM dosage; DTX dosage; usage of immunosuppressive agent; history of prophylactic granulocyte colony stimulating factor (G-CSF); blood biochemical parameters including leukocyte count (×10^4^/μL), neutrocyte count ratio (%), haemoglobin level (g/dL), platelet count (×10^4^/μL), serum aspartate aminotransferase level (IU/L), serum alanine aminotransferase level (IU/L), serum total bilirubin level (mg/dL) and serum creatinine (mg/dL); systolic blood pressure (mmHg); diastolic blood pressure (mmHg); and bleeding. Creatinine clearance (Ccr) was calculated using the Cockcroft–Gault formula: Ccr (mL/min): {140 − age (years)} × weight (kg) × 0.85 (in females)/(serum Cr × 72) [13].

### 2.3. Assessment

RAM + DTX therapy-induced adverse events were graded based on the Common Terminology Criteria for Adverse Events, version 5.0, as follows: grade 3 leukopenia is characterised by a white blood cell count of <2000/µL, grade 3 neutropenia is characterised by a neutrophil count of <1000/µL, grade 3 anaemia is characterised by a haemoglobin level of <8.0 g/dL, grade 3 thrombocytopenia is characterised by a platelet count of <50,000/µL, grade 3 serum aspartate aminotransferase (AST) level is characterised by an AST level of the upper limit of normal (ULN) × 5–20 if the baseline is normal and baseline × 5–20 if the baseline is abnormal, the grade 3 serum alanine aminotransferase (ALT) level is characterised by the ALT level of ULN × 5–20 if the baseline is normal and baseline × 5–20 if the baseline is abnormal, the grade 3 serum total bilirubin (T-Bil) level is characterised by a T-Bil level of ULN × 3–10 if the baseline is normal and baseline × 3–10 if the baseline is abnormal, grade 3 hypertension is characterised by a systolic blood pressure (BP) of ≥160 mmHg or diastolic BP of ≥100 mmHg or the need for two or more medications or stronger treatment than before and grade 3 proteinuria is characterised by urinary protein of ≥3.5 g/24 h or 4 + proteinuria. Data on the adverse events with the highest grade were extracted during the treatment period. The predicted risk factor for the onset grade ≥ 3 neutropenia referred to the previous report, i.e., ≤65 years old, defective performance status, planned RDI of ≥85 and primary G-CSF prophylaxis [14,15].

### 2.4. Statistical Analysis

Normally distributed variables were expressed as mean ± standard deviation. Non-normally distributed variables were expressed as median and interquartile range. The *t*-test and Mann-Whitney U-test were used for parametric and nonparametric pairwise comparisons, respectively. The chi-square test and Fisher’s exact test were used to compare ratios. The Kaplan-Meier method was estimated for the onset period of adverse events and the log-rank test was used to compare the two groups. Cox regression analysis was used to determine risk factors for the onset grade ≥ 3 neutropenia and hazard ratios and 95% confidence intervals (95% CIs) are reported. All statistical analyses were performed using Statistical Package for the Social Sciences (version 24.0; IBM Corporation, Armonk, NY, USA). *p*-values of <0.05 were considered statistically significant. 

## 3. Results

### 3.1. Patient Characteristics

A total of 89 patients were included and no patients were excluded. Among them, 80.9% were male, 69.7% had non-squamous cell carcinomas and 83.1% had stage IV diseases. Different cytotoxic anticancer agents were used as pretreatment in 65 patients (platinum: 46 cases; taxane: 19 cases). In 26 patients who discontinued treatment due to adverse events, the following adverse events occurred (number of patients; multiple events possible): neutropenia in 8, proteinuria in 5, malaise in 4, decreased platelets in 2, fever in 1, pneumonia in 1, skin rash in 1, allergy in 1, phlebitis in 1, liver disorder in 1, bleeding in 1, kidney disorder in 1 and oedema in 1. In addition, 60 patients had grade ≥ 3 neutropenia. In 37 patients (52 times in total) who underwent dose reduction or temporary cessation of anticancer agents, the reasons for dose reduction were neutropenia in 78.4%, adverse events other than neutropenia in 18.9% and unknown in 2.7%.

### 3.2. Adverse Events

Of the grade ≥ 3 adverse events identified in all 89 patients, neutropenia was the most common (67.4%), followed by leukopenia (49.4%) and hypertension (39.3%) (multiple events possible). Data on bleeding events were collected from the subjects’ medical records; however, accurate information cannot be collected from all patients because this was a retrospective study. For mild cases, data during outpatient treatment were more difficult to collect than those during inpatient treatment. Nevertheless, 42 patients experienced adverse events, all of which were grade ≤ 2 events.

### 3.3. Grouping Based on ICI Treatment History

There were 50 patients with a history of ICI treatment (the ICI (+) group) and 39 patients without (the ICI (−) group). The ICIs administered were nivolumab in 15 patients, pembrolizumab in 20, atezolizumab in 7 and durvalumab in 8. No significant differences were observed between the ICI (+) and ICI (−) groups in patient background factors, cancer type and severity, RAM + DTX therapy, blood biochemical parameters and medication (Table 1).

### 3.4. Influence of ICI Treatment History on the Onset of Adverse Events

Nine grade ≥ 3 adverse events that occurred due to RAM + DTX therapy were compared between the two groups (Table 2). Of these, the incidence rates of neutropenia, leukopenia and hypertension were high. However, the incidence of neutropenia significantly differed between the two groups (*p* = 0.002), but no significant differences were found in the incidence of leukopenia or hypertension (leukopenia: *p* = 0.525; hypertension: *p* = 0.516).

### 3.5. Factors Associated with the Occurrence of Grade ≥ 3 Neutropenia

**Figure 1:**
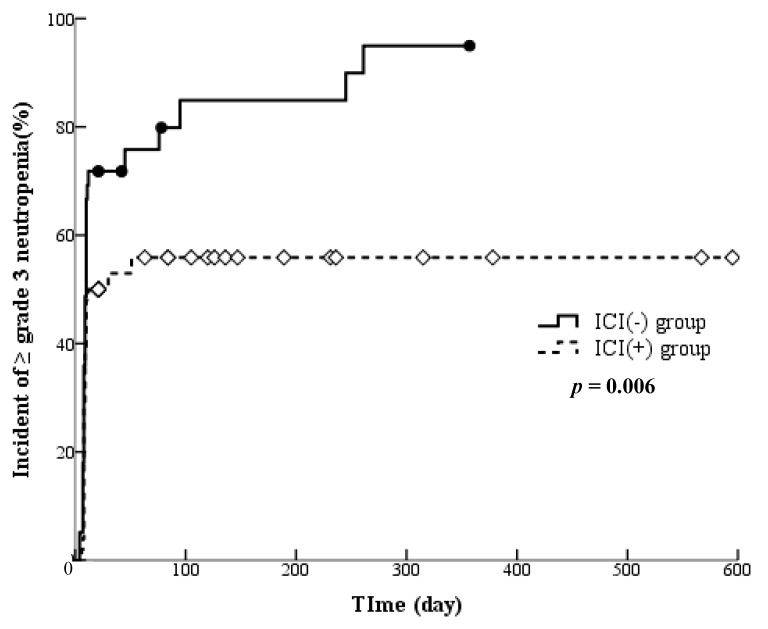
Kaplan-Meier estimates of the incidence of grade ≥ 3 neutropenia in patients with ICI pretreatment history. ICI (+) group: the patient group with ICI pretreatment history (*n* = 50). ICI (−) group: the patient group without ICI pretreatment history (*n* = 39).

The influence of previous ICI treatment on the occurrence of grade ≥ 3 neutropenia is shown as a Kaplan-Meier curve (Figure 1). A clear difference was observed between patients with and without a history of ICI treatment (*p* = 0.006). Moreover, Table 3 displays the results of our search for factors associated with the occurrence of grade ≥ 3 neutropenia. The seven predictors tested were age, gender, disease classification, stage, history of ICI treatment, line of treatment and prophylactic administration of G-CSF. In univariate analysis, two factors were extracted: history of ICI treatment and prophylactic administration of G-CSF; other factors had no influences (history of ICI treatment: *p* = 0.019, prophylactic administration of G-CSF: *p* < 0.001). Then, multivariate analysis using the two extracted factors revealed that both factors were associated with the occurrence of grade ≥ 3 neutropenia (history of ICI treatment: *p* = 0.018, prophylactic administration of G-CSF: *p* < 0.001).

### 3.6. Influences of Pretreatment with Cytotoxic Chemotherapy

The influences of cytotoxic chemotherapy administered as pretreatment were compared between the two groups (Table 4). Cytotoxic anticancer agents were administered as the pretreatment in 49 patients in the ICI (+) group and 37 in the ICI (−) group (*p* = 0.579). The median (IQR) number of days between cytotoxic chemotherapy as the pretreatment and the initiation of RAM + DTX therapy was 137 (28–226) for the ICI (+) and 61 (33–454) for the ICI (−) groups (*p* = 0.754). The pretreatment regimen included platinum in 22 patients in the ICI (+) group and 24 in the ICI (−) group (*p* = 0.135). The pretreatment regimen involved taxane in 10 patients in the ICI (+) group and 9 in the ICI (−) group (*p* = 0.797). A total of 31 patients in the ICI (+) group and 23 patients in the ICI (−) group had a history of radiotherapy (*p* = 0.829). No bias was observed between the two groups regarding a history of pretreatment.

**Figure 2:**
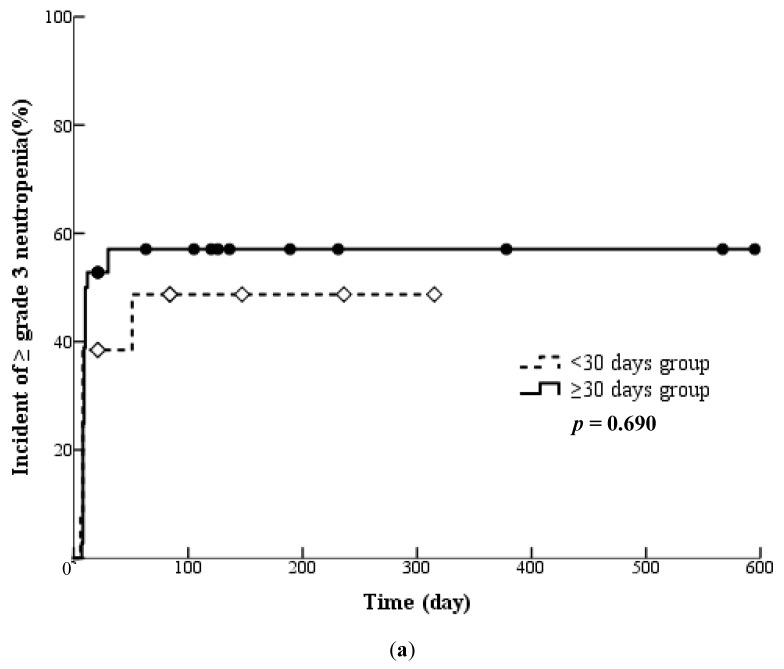

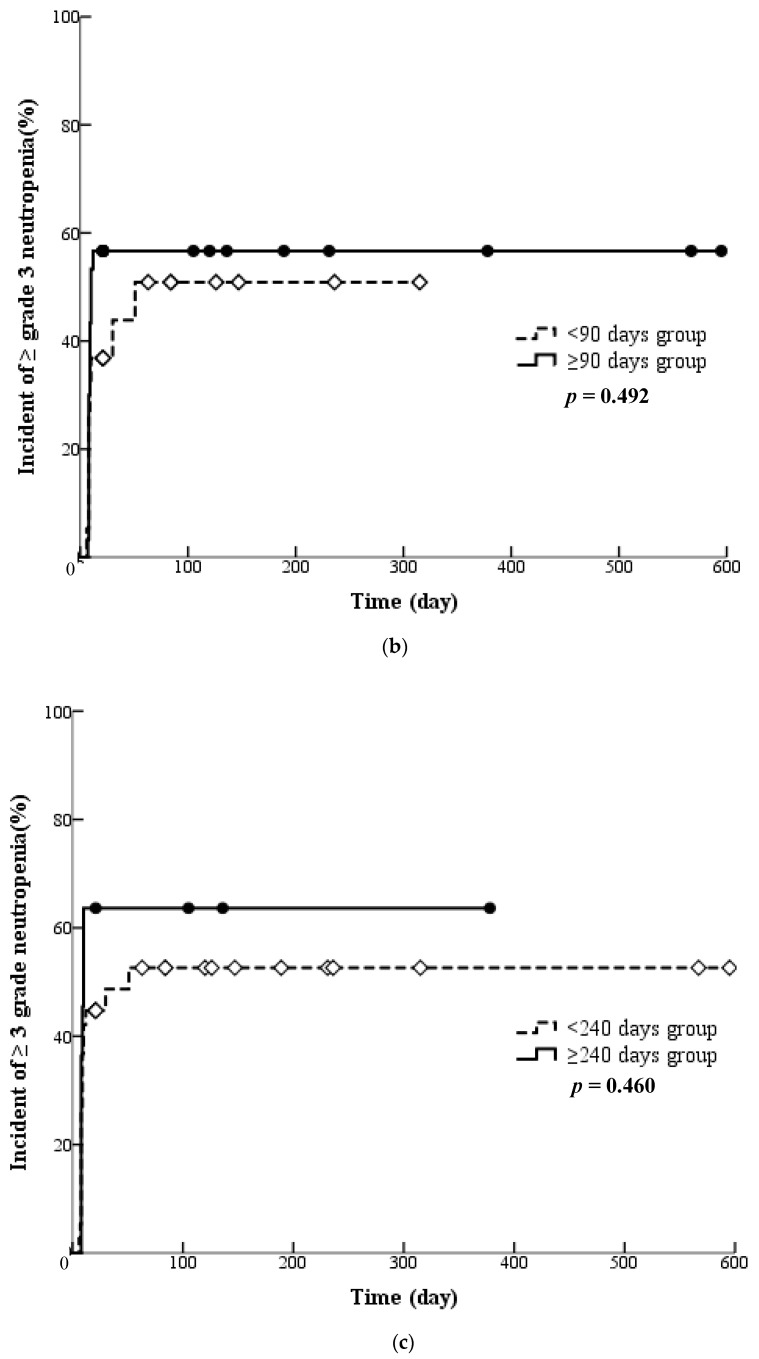
(**a**) Kaplan-Meier curves for the subgroups with an interval from cytotoxic anticancer drug administration of ≥30 days and <30 days in the ICI (+) group. The ≥30 days subgroup: patients who received RAM + DTX therapy ≥30 days post-pretreatment with cytotoxic anticancer drugs (*n* = 36). The <30 days subgroup: patients who received RAM + DTX therapy <30 days post-pretreatment with cytotoxic anticancer drugs (*n* = 13). (**b**) Kaplan-Meier curves for the subgroups with an interval from pretreatment with cytotoxic anticancer drugs of ≥90 days and <90 days in the ICI (+) group. The ≥90 days subgroup: patients who received RAM + DTX therapy ≥90 days post-pretreatment with cytotoxic anticancer drugs (*n* = 30). The <90 days subgroup: patients who received RAM + DTX therapy <90 days post-pretreatment with cytotoxic anticancer drugs (*n* = 19). (**c**) Kaplan-Meier curves for the subgroups with an interval from pretreatment with cytotoxic anticancer drugs of ≥240 days and <240 days in the ICI (+) group. The ≥240 days subgroup: patients who received RAM + DTX therapy ≥240 days post-pretreatment with cytotoxic anticancer drugs (*n* = 11). The <240 days subgroup: patients who received RAM + DTX therapy <240 days post-pretreatment with cytotoxic anticancer drugs (*n* = 38).

In the ICI (+) group, the incidence rates of grade ≥ 3 neutropenia in patients sub-grouped based on the number of days between the day of the last dose of prior cytotoxic chemotherapy and the initiation day of RAM + DTX therapy were analysed. Forty-nine patients were included in the analysis and were divided into two subgroups based on the number of days between pretreatment and the initiation of RAM + DTX therapy as follows: ≥30 (*n* = 36) and <30 days (*n* = 13); ≥90 (*n* = 30) and <90 days (*n* = 19); and ≥240 (*n* = 11) and <240 days (*n* = 38). The incidence of neutropenia was compared between these pairs of subgroups. Significant differences were not found between any of these pairs of subgroups (*p* = 0.690, *p* = 0.492, *p* = 0.460) (Figure 2).

## 4. Discussion

The results of this multicentre retrospective study highlight that previous ICI therapy reduces the incidence of grade ≥ 3 neutropenia after RAM + DTX therapy in patients with NSCLC, regardless of the influences of pretreatment with cytotoxic anticancer agents.

The REVEL study, which demonstrated the additional benefit of RAM + DTX therapy over DTX monotherapy, reported that grade ≥ 3 neutropenia occurred in 49% of all patients undergoing RAM + DTX therapy and was the most frequent grade ≥ 3 adverse event [2]. Furthermore, pretreatment did not affect the occurrence of this event [4]. These two reports are consistent with the results obtained in this study. A phase II trial in Japanese patients with NSCLC showed that the incidence of FN was higher with RAM + DTX therapy (34.2%) than with placebo + DTX (19.8%) [16]. The reason for the higher incidence of FN in Japanese patients with NSCLC than American patients with NSCLC is attributable to the different effects of PTX resulting from genetic differences based on race [17]. Based on these results, FN frequently occurs in Japanese patients and caution should be exercised similarly when performing RAM + DTX therapy to prevent the occurrence of severe neutropenia. Kasahara et al. [18] have reported that prophylactic administration of G-CSF reduced the incidence of FN to <5% after RAM + DTX therapy. Primary prophylaxis with G-CSF has already been established as one way to control neutropenia in Japanese guidelines for the proper use of G-CSF. However, in this study, we would like to focus on the possibility of preventing the occurrence of grade ≥ 3 neutropenia regardless of pretreatment from a different perspective of the influence of previous ICI therapy.

A phase III trial in patients with hepatocellular carcinoma (IMbrave150) has shown that the combined use of atezolizumab, classified as an ICI, and bevacizumab, classified as a vascular endothelial growth factor (VEGF) inhibitor, extended both PFS and OS compared to the use of atezolizumab alone [19]. The combination of VEGF with ICIs has been shown to hold the potential of increasing the efficacy of ICIs and reducing the risk of immune-related adverse effects [20]. This study reported that the incidence of grade ≥ 3 neutropenia with RAM + DTX therapy was significantly reduced in patients with a history of ICI therapy. The aforementioned reports suggest that pretreatment with ICIs had some effect on the efficacy of the subsequently used VEGF inhibitors. Similarly, the phenomenon that a history of ICI therapy reduced the adverse event warrants great attention. The Japanese clinical practice guidelines for NSCLC recommend various therapies as a treatment option for second- and later-line therapy, including RAM + DTX therapy, pemetrexed monotherapy [21], S-1 monotherapy [22], nanoparticle albumin-bound paclitaxel monotherapy [23] and others. Furthermore, DTX monotherapy may be used for the patient with advanced NSCLC previously treated with platinum-based chemotherapy [24,25]. Therefore, although the present study was a multicentre study, the sample size was small, which caused limitations in the analysis.

This study revealed that a history of ICI treatment was associated with a lower incidence of grade ≥ 3 neutropenia, regardless of pretreatment. A study investigating the incidence of grade ≥ 3 neutropenia in Japanese patients with gastric cancer receiving combination therapy with RAM and PTX has reported that pretreatment regimens had no effects [26]. The lack of influence of pretreatment with cytotoxic chemotherapy is consistent with the findings in the present study. However, the above previous report did not include any information about before the ICI treatment and did not consider the influences of pretreatment with ICIs. The results of this study should also be appreciated in this regard. Several previous reports have described the influences of ICI pretreatment on the occurrence of adverse events. Kotake et al. [10] have reported that immediate previous nivolumab therapy increased the incidence of interstitial pneumonia in patients treated with osimertinib. Other relevant findings reported include an increased risk of hepatotoxicity associated with crizotinib administered after ICI therapy [27]. Meanwhile, a study has shown that the frequency of peripheral sensory neuropathy (all grades), grade ≥ 3 leukopenia or neutropenia with nab-PTX monotherapy did not significantly differ with or without ICI pretreatment [28].

The median age of the study participants was 69 years and the gender was predominantly male. Thus, in the 2019 Cancer Statistics in Japan conducted by the Japanese Foundation for Promotion of Cancer Research, the age and sex distribution of participants were similar to that of the Japanese population [29]. Meanwhile, this study had some limitations. Because this was a retrospective study using the data collected from electronic medical records, data on some variables, such as smoking history, bleeding events and body temperature, were missing and their effects cannot be accounted for. Despite these limitations, the study results are commendable in that the following significant events were revealed: (1) the analysis of the interval from the administration of cytotoxic anticancer agents in the pretreatment showed no significant differences between the two subgroups, ruling out the influences of bone marrow suppression due to pretreatment, and (2) the results of the multivariate analysis demonstrated that a history of ICI treatment affected the incidence of grade ≥ 3 neutropenia.

Several studies have reported the effects of ICIs on RAM + DTX therapy. The administration of ICIs before RAM + DTX therapy has been shown to improve the efficacy of RAM + DTX therapy [30,31,32,33]. Tanizaki et al. [34] have reported that in the group with prior ICIs, the objective response rate (ORR), disease control rate (DCR) and PFS were significantly higher than those without prior ICIs, whereas there were no significant differences in PFS between the groups with and without a cytotoxic agent alone, bevacizumab and tyrosine kinase inhibitor. Others have reported that patients pretreated with ICIs responded well to RAM + DTX therapy, regardless of PD-L1 expression [35]. Brueckl et al. [36] have reported a possible synergistic effect for RAM + DTX therapy after the ICI administration regardless of NSCLC histology. Furthermore, Ishida et al. [37] compared DTX therapy and RAM + DTX therapy after the ICI therapy and concluded that RAM + DTX therapy prolonged the ORR compared to single-agent chemotherapy by the action of the anti-VEGFR2 antibody RAM to overcome tolerance after long-term chemoimmunotherapy by improving the hypoxic TME. Yamaguchi et al. [38] have reported prolonged PFS in patients with NSCLC who underwent immunotherapy for at least 180 days before RAM + DTX therapy. Thus, a history of ICI treatment appears to enhance the efficacy of subsequent combination therapy with RAM. Yi et al. [39] have reported that the anti-angiogenic property and ICI therapy mutually increased the efficacy. Specifically, the anti-angiogenic property increases the ratio of anti/pro-tumour immune cells and reduces the expression of multiple immune checkpoints, and in ICI therapy, the normalisation of blood vessels reduces the required ICI doses because of improved drug delivery, decreasing the risk of adverse events. Moreover, Lin et al. [40] have reported that low-dose PTX increased the PD-L1 level and CD8+ T-cell percentage in the tumour immune microenvironment. The events observed in this study may be explained by these mechanisms. Matsuzawa et al. [41] have reported that RAM + DTX therapy demonstrated encouraging antitumor activity with a manageable safety profile in patients who have progressed on front-line ICIs plus platinum-based chemotherapy. Several phase III studies have shown the survival superiority of platinum-based chemotherapy combined with ICIs to platinum-based chemotherapy alone [42,43,44]. Thereafter, ICIs plus platinum-based chemotherapy as a front-line treatment became frequently used after recent guideline revisions [45]. The influence of ICIs on the development of severe neutropenia caused by RAM + DTX therapy after front-line ICIs plus platinum-based chemotherapy cannot be explained based on this study. 

In this study, we found that previous treatment with ICIs reduced the incidence of grade ≥ 3 neutropenia after RAM + DTX therapy in patients with NSCLC, regardless of the influences of pretreatment with cytotoxic anticancer agents. Currently, indications for ICIs are being expanded to multiple organs. Therefore, the benefits of pretreatment with ICIs are worth considering for patients in whom combination therapy with angiogenesis inhibitors is an option for future treatment.

## 5. Conclusions

Regardless of pretreatment history with cytotoxic anticancer agents, previous ICI administration significantly reduces the incidence of severe neutropenia caused by subsequent RAM + DTX therapy. This multicentre study was able to break the limitations of single-centre studies that were carried out in the past, which demonstrated the following: (1) Cox regression analysis raised the reliability of identification of the factors associated with the occurrence of severe neutropenia caused by RAM + DTX therapy, and (2) the validation results of pretreatment with cytotoxic chemotherapy raised the reliability of verification of the influence of previous ICI therapy on the development of severe neutropenia caused by RAM + DTX therapy.

## Figures and Tables

**Table 1:** Comparison of the characteristics between the ICI (+) and ICI (−) groups.

	ICI (+) Group (*n* = 50)	ICI (−) Group (*n* = 39)	*p*-Value
Age (years)	67 (61–72)	68 (65–74)	0.097
Body weight (kg)	58.5 (53.0–67.5)	62.0 (55.2–68.0)	0.307
Body surface area (m^2^)	1.60 (1.52–1.73)	1.63 (1.53–1.72)	0.869
Male/female (n)	41/9	31/8	0.765
Performance status: 3 or 4 (n)	1	1	1.000
WHO classification (n)			
Squamous cell carcinoma	13	7	0.373
Non-squamous cell carcinoma	32	30	
Unknown	5	2	
Stage (n)			
III	9	5	0.480
IV	40	34	
Unknown	1	0	
Chemotherapy			
Number of courses of RAM + DTX therapy	4.0 (2.0–7.8)	4.0 (2.0–5.5)	0.582
Prophylactic G-CSF administration (%)	40.0	43.6	0.733
RDI			
RAM	96.4 ± 5.2	95.5 ± 5.3	0.421
DTX	91.5 ± 8.7	91.9 ± 8.8	0.825
Blood biochemical parameter at baseline			
WBC (/μL)	6250 (5225–9100)	6500 (5500–8300)	0.960
AST (IU/L)	23 (16–29)	23 (20–28)	0.571
ALT (IU/L)	16 (11–26)	16 (11–22)	0.859
T-Bil (mg/dL)	0.43 (0.30–0.60)	0.48 (0.40–0.60)	0.289
Ccr (mL/min)	69.0 (54.3–88.8)	66.2 (55.9–79.9)	0.350
Medication			
Immunosuppressives	10	6	0.782

WBC, white blood cell; AST, aspartate aminotransferase; T-Bil, total bilirubin; Ccr creatinine clearance.

**Table 2:** Comparison of the ratio of grade ≥ 3 adverse events between the ICI (+) and ICI (−) groups.

Adverse Event	Number of Patients		*p*-Value
	ICI (+) Group (*n* = 50)	ICI (−) Group (*n* = 39)	
Leukopenia	23	21	0.525
Anaemia	5	3	0.732
Thrombocytopenia	2	3	0.650
AST	3	1	0.628
ALT	2	0	0.502
T-Bil	0	0	1.000
Hypertension	18	17	0.516
Proteinuria	3	4	0.695
Neutropenia	27	33	0.002

**Table 3:** Factors associated with the occurrence of grade ≥ 3 neutropenia.

Factor	Univariate Analysis Hazard Ratio (95% CI)	*p*-Value	Multivariate Analysis Hazard Ratio (95% CI)	*p*-Value
Age	1.012 (0.981–1.045)	0.446		
Male	0.989 (0.512–1.913)	0.989		
Squamous cell carcinoma	1.302 (0.720–2.353)	0.383		
Stage IV	1.465 (0.693–3.095)	0.317		
Usage history of immune checkpoint inhibitor	0.540 (0.323–0.903)	0.019	0.538 (0.322–0.900)	0.018
Course number of RAM + DTX therapy	0.968 (0.803–1.168)	0.736		
With prophylactic G-CSF	0.263 (0.139–0.499)	<0.001	0.264 (0.140–0.499)	<0.001

**Table 4:** Pretreatment.

	ICI (+) Group (*n* = 50)	ICI (−) Group (*n* = 39)	*p*-Value
Pretreatment with cytotoxic anticancer drugs	49	37	0.579
Interval (days) from pretreatment with cytotoxic anticancer drugs	137 (7–79)	61 (22–1637)	0.754
Medical history of immediate prior chemotherapy			
Chemotherapy			
Platinum	22	24	0.135
Taxane	10	9	0.797
Medical history			
Radiotherapy	31	23	0.829

## Data Availability

Data are contained within the article.
